# Novel FXa Inhibitor Identification through Integration of Ligand- and Structure-Based Approaches

**DOI:** 10.3390/molecules22101588

**Published:** 2017-09-22

**Authors:** Carlos F. Lagos, Gerardine F. Segovia, Nicolás Nuñez-Navarro, Mario A. Faúndez, Flavia C. Zacconi

**Affiliations:** 1 Department of Endocrinology, School of Medicine, Pontificia Universidad Católica de Chile, Lira 85, Santiago 8330074, Chile; cflagos@uc.cl; 2 Facultad de Ciencia, Universidad San Sebastián, Campus Los Leones, Lota 2465, Providencia, Santiago 7510157, Chile; 3 Departamento de Química Orgánica, Facultad de Química, Pontificia Universidad Católica de Chile, Av. Vicuña Mackenna 4860, Macul, Santiago 7820436, Chile; gfsegovi@gmail.com (G.F.S.); nrnunez@uc.cl (N.N.-N.); 4 Departamento de Farmacia, Facultad de Química, Pontificia Universidad Católica de Chile, Av. Vicuña Mackenna 4860, Macul, Santiago 7820436, Chile; mfaundeza@uc.cl; 5 Centro de Investigación en Nanotecnología y Materiales Avanzados, CIEN-UC, Pontificia Universidad Católica de Chile, Av. Vicuña Mackenna 4860, Macul, Santiago 7820436, Chile

**Keywords:** factor Xa, virtual screening, shape-based screening, protein-ligand docking, enzyme inhibitors, blood coagulation cascade

## Abstract

Factor Xa (FXa), a vitamin K-dependent serine protease plays a pivotal role in the coagulation cascade, one of the most interesting targets for the development of new anticoagulants. In the present work, we performed a virtual screening campaign based on ligand-based shape and electrostatic similarity search and protein-ligand docking to discover novel FXa-targeted scaffolds for further development of inhibitors. From an initial set of 260,000 compounds from the NCI Open database, 30 potential FXa inhibitors were identified and selected for in vitro biological evaluation. Compound **5** (NSC635393, 4-(3-methyl-4*H*-1,4-benzothiazin-2-yl)-2,4-dioxo-*N*-phenylbutanamide) displayed an IC_50_ value of 2.02 nM against human FXa. The identified compound may serve as starting point for the development of novel FXa inhibitors.

## 1. Introduction

Anticoagulation therapy remains the cornerstone for the prevention and treatment of progressive or recurrent thromboembolic disorders, which are among the major causes of morbidity and mortality [1,2]. Anticoagulants are effective in the treatment of acute coronary syndrome, as well as angina pectoris [3]. Prothrombinase complex is involved in the conversion of prothrombin (FII) into thrombin (FIIa), the first step of the coagulation cascade. Thrombin is produced by activated factor X (FXa) mediated cleavage of two sites on prothrombin, activity that is enhanced by FXa binding to activated FV (FVa), thus creating the prothrombinase complex [4]. Furthermore, FXa lies at the convergence of the extrinsic and intrinsic coagulation pathways and thereby regulates amplification of the coagulation cascade, making this enzyme an attractive target for anticoagulant drug therapies [5].

**Figure 1:**
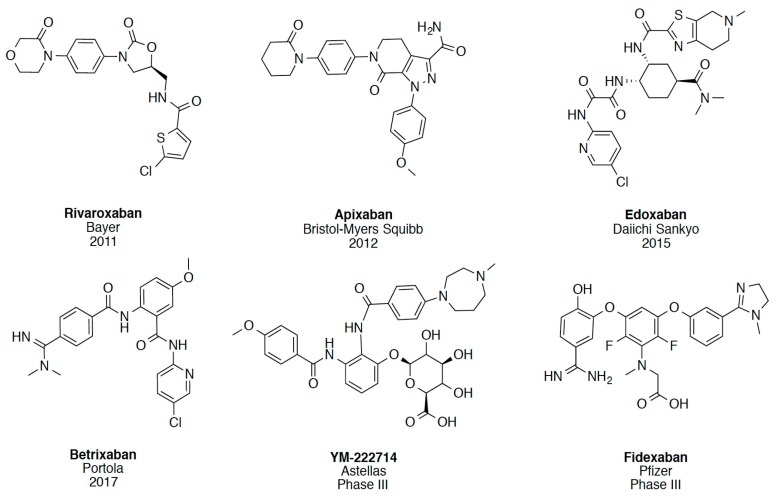
Currently approved and investigational FXa inhibitors.

In recent years, several direct FXa inhibitors have entered to the clinic, and others are in active development (Figure 1). Although these new oral anticoagulants are more efficacious than warfarin for the prevention of stroke and systemic embolism in patients with atrial fibrillation, the absence of a reversal agent is a barrier to more widespread use of these agents [6,7,8]. As with all anticoagulants, a serious adverse event of concern with FXa inhibitors is the risk of major uncontrolled bleeding and life-threatening bleeding events [9]. Additionally, recent studies have shown that Rivaroxaban and Apixaban discontinuation could result in thromboembolic events and the use of Rivaroxaban associated with warfarin increases the risk of major bleeding in non-valvular atrial fibrillation patients [10,11,12]. Therefore, the search for novel FXa inhibitor chemotypes with different properties is necessary.

**Figure 2:**
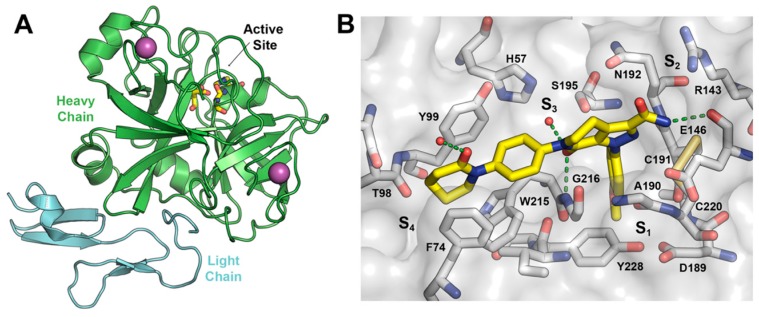
(**A**) Schematic representation of FXa protein structural domains showing the location of the binding site and ions within the heavy chain (green cartoon) and the light chain (light blue cartoon); (**B**) The X-ray crystal structure of Apixaban (yellow carbons) in complex with human FXa (PDBid 2P16). Essential amino acids and binding pockets are indicated; hydrogen bonds between Apixaban and FXa residues and structural waters are shown as green dotted lines.

FXa has two chains of amino acids linked by a disulfide bridge. The heavy chain consists of 303 amino acids and the light one of 139 amino acids. The catalytic triad is present in the heavy chain and comprises residues Ser195, His57, and Asp102 (Figure 2A). The hydrogen bonding network between Asp102, His57, and Ser195, named the charge relay system, activates Ser195 for nucleophilic attack [13]. The binding pockets in FXa follows the Schechter and Berger nomenclature [14].

The crystal structure of the FXa-Apixaban complex shows the typical curved shape of FXa inhibitors (Figure 2B), wherein the aromatic 4-methoxyphenyl group binds towards the S_1_ subsite pocket defined by residues Trp215 (main-chain atoms) and Gly216 on one side and Ala190, Cys191, and Gln192 on the other side. The S_1_ pocket bottom is lined by Asp189 and the side chain of Tyr228. The S_4_ binding pocket is an aromatic box formed by the side chains of Tyr99, Phe174, and Trp215 (side chain) where the phenylpiperidinone group accommodates. To date, more than 270 crystal structures of FXa are available at the protein data bank [15,16], of which 150 have a co-crystallized small-molecule ligands, thereby providing a rich source of structural information for the design of novel enzyme inhibitors, including Apixaban and Rivaroxaban, encouraging us to perform a virtual screening (VS) campaign for the discovery of novel FXa inhibitor chemotypes.

Virtual screening is a complementary approach to high-throughput screening (HTS), and has been successfully adopted. This approach reduces the number of compounds that need to be tested from large chemical libraries [17,18]. Virtual screening can be classified into two categories: structure-based virtual screening and ligand-based virtual screening [17,19]. Considering the inherent complementarities of structure-based virtual screening and ligand-based virtual screening, the technique has been successfully employed for the discovery of novel FXa inhibitors [20,21,22,23,24].

In this work, we illustrate a report of a virtual screening workflow based on a combination of ligand shape/electrostatic similarity screening techniques with docking to identify novel scaffolds as potential FXa inhibitors and the corresponding in vitro biological assays of the selected molecules.

## 2. Results and Discussion

### 2.1. Protein-Ligand Complexes Selection and Ligand Clustering

As the first step of our approach, we searched the Protein Data Bank (PDB) for human FXa available structures. This search rendered 270 FXa crystal structures, from which 144 have co-crystallized ligands, including crystallization moieties and ions, and 127 of them were solved in complex with small molecules FXa inhibitors. A substructure search using the phenyloxomorphonilo or phenyloxopiperidine scaffolds present in Apixaban and Rivaroxaban as a query, renders a set of 20 FXa-ligand complexes, which was compiled and their structures retrieved from the PDB. We have selected these fragments as starting point because along with the S_1_ site, S_4_ represents by far the most important binding region for factor Xa inhibitors [25]. These groups have shown to be as the most important group for potency in these two drugs that were the first approved FXa inhibitors [26,27].

**Figure 3:**
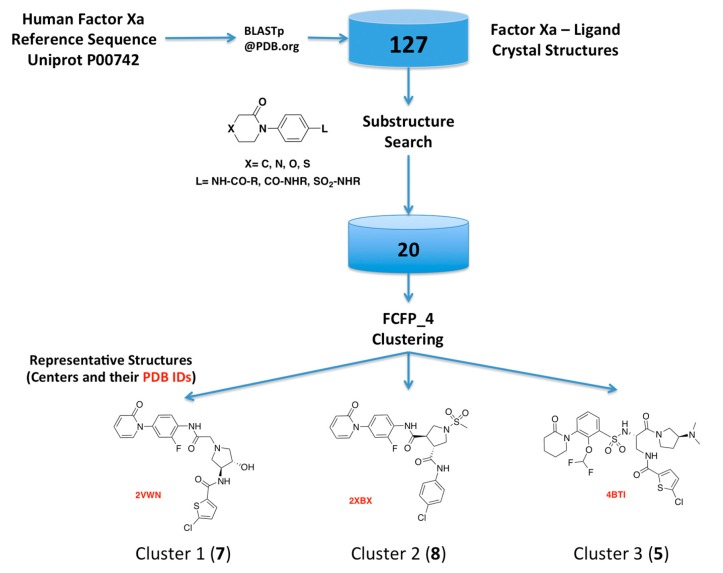
Schematic overview of the procedure used for selecting the FXa-ligand complexes.

Hits from the ligand substructure search were clustered using the FCFP_4 (functional-class extended-connectivity fingerprint count up to diameter 4) set renders three major clusters (Figure 3) according to the Tanimoto distance, defined as 1 − (N_A&B_/N_A_ + N_B_ − N_A&B_), wherein N_A_ represents the number of “on” features (bits) in structure A. N_B_ represents the number of “on” features (bits) in structure B. N_A&B_ represents the number of “on” features (bits) common to both fingerprints A and B. The hashed binary chemical fingerprint of a molecule is a bit string (a sequence of “0” and “1” digits) that contains information on the structure. FCFPs represent circular atom neighborhoods and produce fingerprints of variable length, which are further abstracted in that instead of indexing a particular atom in the environment, they index that atom’s role [28,29].

Proteins for each cluster were edited, and only one monomer was used if multiple chains were available for the coordinates, the valence checked, and atom-type assigned using the CHARMM22 force-field (Accelrys Discovery Studio v2.1, Accelrys Inc., San Diego, CA, USA). All structures within each cluster were structurally aligned against the protein corresponding to the cluster center and the root-mean square deviation (RMSD) for the α-carbon for all residues and residues within 6 Å from the measured center of mass of all co-crystallized ligands are shown in Table 1.

The alpha-carbon root-mean square deviation (Cα-RMSD) against the low-resolution FXa-ligand structure on each cluster ranged from 0.120 to 0.530 Å, while the all-atom RMSD within 6 Å from the center of mass of all aligned ligands ranged from 0.269 to 1.019 Å, indicating that, within clusters, proteins have no significant conformational differences in the binding sites in comparison with the reference structure.

### 2.2. Virtual Screening and Compound Selection

**Figure 4:**
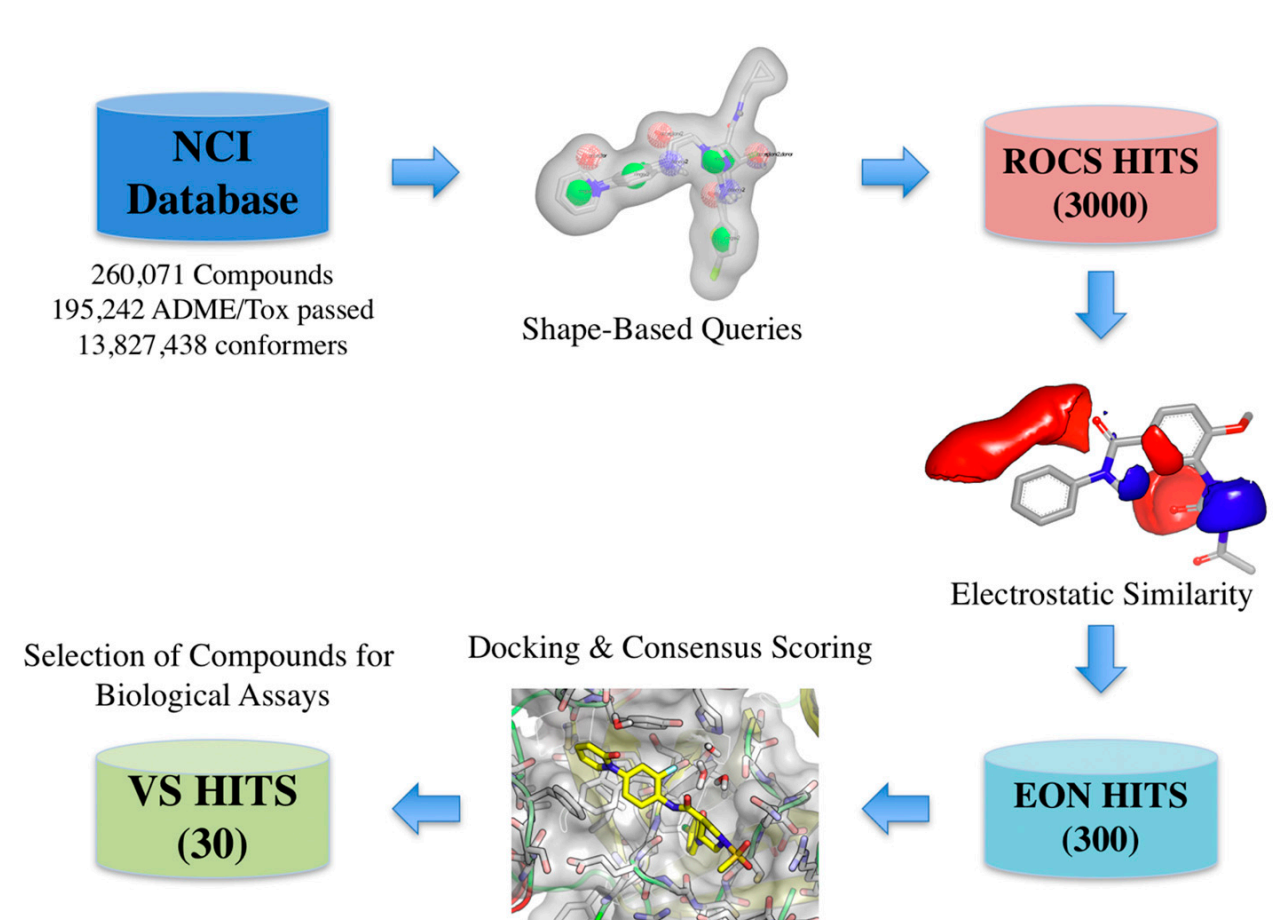
Schematic overview of the virtual screening workflow and the procedure used for selecting the virtual screening hits whose bioactivity was experimentally tested. The number of compounds that passed each step and the programs used are shown. From an initial set of near 260,000 compounds, 300 compounds were identified as putative FXa inhibitors by the virtual screening workflow. Thirty of these compounds were selected for in vitro testing.

The virtual screening workflow used consisted in: (a) filtering the library using the shape- and electrostatic-based queries derived from each cluster, and (b) screening the hits using docking and consensus scoring to select the compounds for biological assays (Figure 4).

The molecular probes are regular molecules, but have been chosen to represent common shapes of drug-like molecules. The release 3 (September 2003) version of the OpenNCI database containing 260,071 structures in SDF format was retrieved from NCI/CAAD group website [35]. The library was standardized and filtered by ADME/Tox constraints rendering a database of 195,242 compounds. Multiple conformations for each compound in the database were generated using OMEGA v2.5.1.4 (OpenEye Scientific Software, Santa Fe, NM, USA) using the default parameters [36,37].

#### Shape and Electrostatic-Based Queries Development and Validation

**Figure 5:**
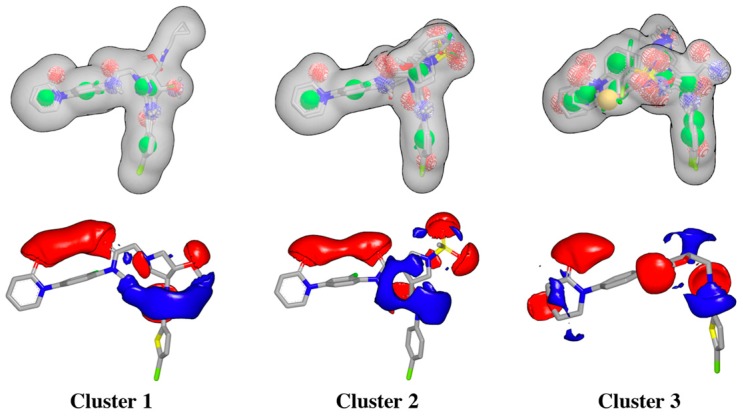
Schematic overview of ROCS shape-based queries and electrostatic grids based on the center ligand corresponding to each cluster.

Rapid Overlay of Chemical Structures (ROCS) is a highly efficient shape comparison application which is based on the principle that molecules will form similar shapes if their volumes overlay well [38,39]. After protein structure superposition, the ligand clustering procedure identified three main clusters according to the FCFP_4 fingerprints, and ligands within each cluster were very similar, as estimated by the Tanimoto coefficients (Table 1). The 3D alignment of the ligands for each cluster was used to generate the shape-based queries, which were validated using a library of decoys, and the ligand corresponding to cluster center was selected for generating the electrostatic grids query for each cluster (Figure 5).

**Figure 6:**
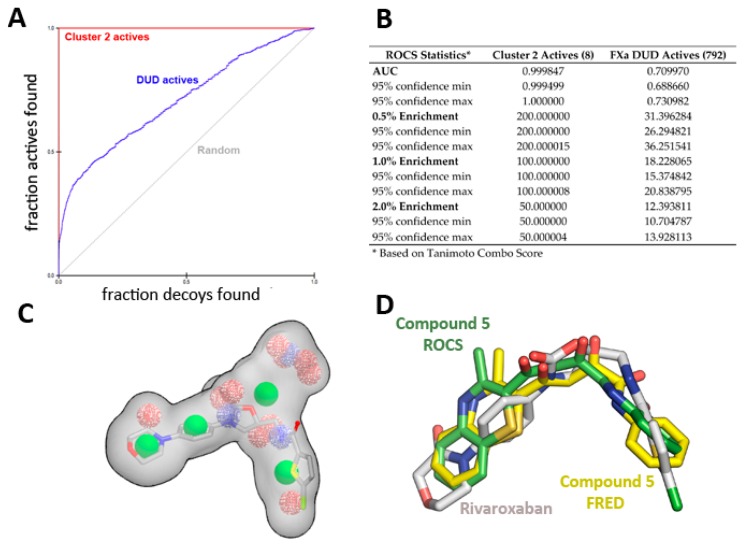
ROCS shape-based query validation statistics for Cluster 2. (**A**) ROC plot and (**B**) validation statistics using the active ligands of cluster 2 or the FXa actives from the DUD-E database. (**C**) Superposition of Rivaroxaban over the cluster 2 shape-based query, and (**D**) Comparison of crystallographic position of Rivaroxaban (PDB id 2W26) with the position obtained for compound **5** with ROCS and FRED runs.

The enrichment curve plots the number of active compounds recovered versus the proportion of the database screened. The AUC (area under the curve of the ROC plot) is defined as the probability that a randomly-chosen active compound has a higher score than a randomly-chosen inactive compound. As shown in Figure 6, the AUC of the probability obtained for the hypotheses from cluster 2 is higher than 99% at ±95% confidence (Figure 6A,B), suggesting the shape query hypothesis can be considered highly selective when using the actives that correspond to each cluster.

However, when using a more diverse collection of FXa inhibitors from the extended database of useful decoys (DUD), the AUC probability falls near 70%. A similar trend is observed for clusters 1 and 3, displaying AUCs of 0.74 and 0.79, respectively (see Supplementary Figure S1 for ROC plot and statistics). Cluster 3 has the highest AUC, suggesting it is the most selective query over the entire database search. Although most actives rank higher than most of the decoy molecules, the hypotheses are considered mildly selective, and only 5% of the top scoring solutions per cluster were retrieved (1000 each).

After the generation of the shape/electrostatics-based queries, the virtual screening protocol was applied to the National Cancer Institute (NCI) Developmental Therapeutics Program (DTP) database. The shape similarity between the screened compounds and each cluster was evaluated by the Tanimoto_Combo score method, which consists of the Tanimoto coefficient and the score retrieved from the ROCS color force field, which represents the structural complementarity between the template and the screened molecules. Briefly, the ROCS color force field describes one molecule by the spatial arrangement of six types of chemical features such as: hydrogen-bond donors, hydrogen-bond acceptors, hydrophobic, anions, cations, and rings. The Tanimoto_Combo score ranges from 0 to 2, the higher the score, the more similar a given compound is to the query. Next, EON was used to calculate the electrostatic similarity between the ROCS overlay hits and the query in the form of an Electrostatic Tanimoto (ET) score. The ET_Combo Score that takes into account both shape match and ET match, which was used for the selection of the molecules for docking ranges from 0 to 2. Ten percent of the scoring solutions per cluster were retrieved in this step.

Docking of multiple molecular probes into the active site creates the shape potential, from which the inner and outer contours are generated [40]. Virtual screening of the generated NCI conformer database was performed using the FRED v3.2.0.2 program (OpenEye Scientific Software, Santa Fe, NM, USA) using the ChemGauss3 scoring function [41,42]. Although docking retains important liabilities (it cannot calculate affinities accurately nor even reliably rank order high-scoring molecules), it can often distinguish likely from unlikely ligands, often with hit rates above 10% [43].

The top binding mode for the best 1000 scoring compounds on each protein binding site were selected according to a consensus score using the ChemGauss3, OEChemScore, and PLP scoring functions [44]. The corresponding shape-based hypothesis previously developed for each cluster was included as a filter during the docking procedure [42]. The top 100 scoring compounds on each docking run were visually analyzed. An overlap analysis of obtained solutions using the Tanimoto similarity metric was performed with InstantJChem v5.9 (ChemAxon Inc., Budapest, Hungary) to select 30 compounds that after evaluating their availability were requested and obtained from to the Developmental Therapeutic Program at NCI-NIH (DTP/NIH) [45].

### 2.3. Biological Evaluation of FXa Inhibition

All compounds were evaluated in vitro for their FXa enzyme inhibitory activity using Rivaroxaban as the positive control. Table 2 summarizes the FXa inhibitory activity for selected compounds and the identified hit compound **5** (NSC635393). The molecular structures, National Service Center codes (NSC), FRED docking scores, and estimated binding free energies of the selected binding modes for each compound are given in Table S1—Supporting Information.

The assay showed that several of the novel compounds exhibited inhibitory activity against FXa at 10 μM, but only compound **5** displayed FXa inhibition higher than 50% at 1 μM. The maximal effect of FXa inhibition for compound **5** was 58% at 10 nM, while the maximal effect for Rivaroxaban was 81% at 10 nM. It is noteworthy that compound **5** (NSC635393), has the best ROCS ranking score of the shape-based hypothesis for its cluster.

**Figure 7:**
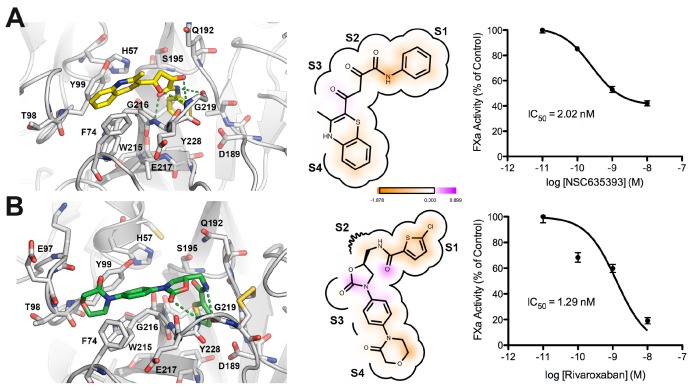
Comparison between the predicted binding mode and in vitro FXa inhibition for (**A**) compound **5** (NSC635393) and (**B**) Rivaroxaban.

Figure 7 shows a comparison between the predicted bound form of compound **5** (NSC635393) and Rivaroxaban. Both molecules adopted a rather similar binding mode in which the phenyl group was found to be oriented toward the S_1_ subsite as the 5-chlorothiophenyl-carbonyl group of Rivaroxaban, while the amide group forms hydrogen bonds with atoms in the main carboxyl group chain of Gly219 and NH group of Gly216. The dioxo group of **5** (NSC635393) forms a strong hydrogen bond with the oxygen atom of the carbonyl group belonging to Gly216 at the S_2_–S_3_ subsites. The 3-methyl-benzothiazine moiety is oriented to the S_4_ subsite flanked by the three following aromatic residues: Phe74, Tyr99, and Trp215. Dose response curves indicate an IC_50_ value of 2.02 nM for compound **5** (NSC635393) and 1.29 nM for Rivaroxaban.

**Figure 8:**
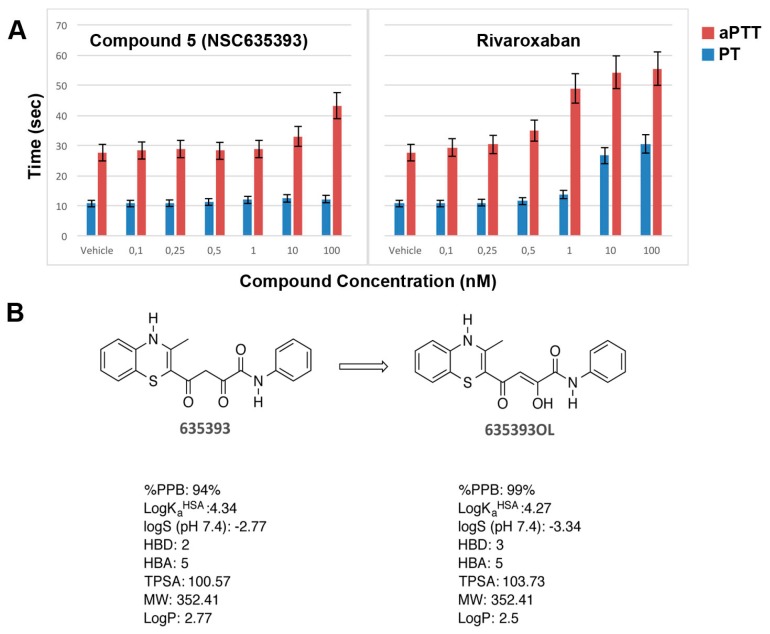
(**A**) In vitro aPTT and PT of compound **5** (NSC635393) and Rivaroxaban using human plasma; (**B**) Tautomerization of compound **5** (NSC635393) to its enol form, with predicted physicochemical and plasma protein binding properties.

In vitro anticoagulation efficacy of compound **5** (NSC635393) and Rivaroxaban was evaluated by activated partial thromboplastin time (aPTT) and prothrombin time (PT) using human plasma (Figure 8A).

As shown in Figure 8, compound **5** (NSC635393) prolonged only aPTT without effect on PT up to 10 nM, while Rivaroxaban simultaneously prolonged aPTT and PT in a dose-dependent manner.

Since the predicted unbound fraction in human plasma of compound **5** (NSC635393, 6%) was higher than that of compound **5** enol tautomer (1%), it is plausible that PT results are consistent with the free drug hypothesis or, alternatively, that compound **5** (NSC635393) may target other coagulation factors, which selectively block the intrinsic pathway at the assayed dose levels.

A structure search on the ChEMBL database [46,47] indicate that compound **5** (NSC635393) has been assayed 53 times in tumor cell line growth inhibition assays, and found to be inactive in all of them. Structural alerts suggest a potential pan-assay interference compounds (PAINS) fragment [48,49], in particular a hetero thioketone moiety present in compound **5**. However, compound **5** did not present a fluorescence quench at the assayed wavelength. On the other hand, detailed analysis of PAINS alerts suggested that researchers should be cautioned about using the current PAINS alerts as reliable indicators of nonspecific pan-assay interference [50].

## 3. Materials and Methods

### 3.1. FXa-Ligand Structure Retrieval and Standardization

Reference sequence for human FXa was obtained from Uniprot database (entry code P00742). The protein databank PDB advanced search tool was used to BLAST the sequence and identify all available human FXa crystal structures. All small co-crystallized ligands were retrieved in SMILES format for the substructure search, and as a pdb file for further clustering by chemical similarity according to the Tanimoto distance by using the FCFP_4 (functional-class extended-connectivity fingerprint count up to diameter 4) fingerprint descriptors set available within Discovery Studio v2.1 suite (Accelrys Inc., San Diego, CA, USA).

Protein preparation, editing, atom typing, and structural alignment were performed using Accelrys Discovery Studio v2.1 (Accelrys Inc., San Diego, CA, USA). Co-crystallized ligands plus crystallographic waters found within 4 Å from the center of mass of the ligand structure were retained. Visual inspection and manual modification of ligand structures valences were performed to ensure a proper state in the protein, and the 20 crystal structures of the selected human FXa-ligand complexes structures were aligned. After superimposition, the Cα-RMSD against the lower resolution structure was calculated. Additionally, an all atom RMSD calculation, including only residues within 6 Å from the center of mass of all crystallized ligands, was also performed. All FXa-ligand complexes atoms were typed with the CHARMM force field for further docking experiments [51].

### 3.2. Shape-Based Query Generation, Validation, and Screening with Electrostatic Similarity Filtering

First, the 3D alignment of the ligands was used to generate shape-based queries with vROCS v3.2 (OpenEye Scientific Software, Santa Fe, NM, USA). The queries were validated using the active compounds from each cluster and also using the FXa inhibitors dataset from the extended database of useful decoys (DUD-E) database [52], that contains 792 actives and 24,017 decoys molecules. The NCI database was retrieved from the DTP website, filtered for counter ions and known toxic moieties and standardized by using the InstantJChem v5.9 (ChemAxon Inc., Budapest, Hungary) and a multiple conformer database was generated by OMEGA v2.5.1.4 (OpenEye Scientific Software, Santa Fe, NM, USA) Shape-based screening was performed by using ROCS v3.2 and a secondary electrostatic similarity search was performed by using EON v2.2.0.5 (OpenEye Scientific Software, Santa Fe, NM, USA).

### 3.3. Docking of Primary Shape/Electrostatic-Based Hits

Receptor binding sites were prepared with Make_Receptor GUI and docking calculations were performed by using FRED v3.2.0.2 (OpenEye Scientific Software, Santa Fe, NM, USA). The AM1BCC charges for ligands and AmberFF99sb for proteins were assigned by using QUACPAC v1.6.3.1 (OpenEye Scientific Software, Santa Fe, NM, USA). FRED docks multiconformer molecules into a single receptor by using an exhaustive search that systematically searches rotations and translations of each conformer of the ligand within the active site [38,40]. The Chemgauss3 scoring function entails these interactions: steric, hydrogen bond, metal–ligand, ligand, and protein desolvation. Each interaction was described by a base function that was smoothed by convolution with a Gaussian function [48]. The surviving poses were scored with a scoring function (default = Chemgauss3), and the top 100 (default) poses were passed to optimization. In optimization, a systematic solid body optimization was done by rigidly rotating and translating the poses at half the step size used in exhaustive docking. Chemgauss3 (default) was used in this step to score the poses during optimization. The poses then go to the consensus structure, in which the poses with the top consensus scores (default = PLP, Chemgauss3, and OEChemscore) were retained, and all other poses were discarded. For the final selection, we used the Chemgauss4 score, a modification of the Chemgauss3 scoring function with improved hydrogen bonding and metal chelator terms. Finally, the top ranked binding modes for each compound were minimized by using the CHARMM22 force field in Discovery Studio v2.1 (Accelrys Inc., San Diego, CA, USA). The minimization protocol allowed the side chains of residue located within 6 Å from the mass centroid of all docked ligands, by using the conjugate gradient algorithm until convergence criteria of 0.001 kcal/mol/Å for the RMS of the energy gradient. Table 2 summarizes the energy evaluation performed for each obtained complex by using the PLP, LigScore, PMF, and LUDI scoring functions, and the consensus scoring available with Discovery Studio v2.1 (Accelrys Inc., San Diego, CA, USA).

### 3.4. Inhibition of FXa In Vitro

The selected compounds were measured in vitro for their factor Xa inhibition using chromogenic substrates. In a first trial the 30 compounds obtained from the developmental therapeutic program at NCI were dissolved in dimethyl sulfoxide (DMSO) at a concentration of 100 mM and then diluted in DMSO to 100 μM. The enzymatic assay was prepared according to manufacturer’s instructions (SensoLyte^®^ Rh110 Factor Xa Assay Kit *Fluorometric*, Anaspec, Fremont, CA, USA). Inhibition control 1 mM was diluted 1:10 with the prepared buffer 1X obtaining the 0.1 mM inhibition control. The reagents containing compound dilutions, buffer and enzyme were mixed, centrifuged, and incubated for 30 min at 37 °C in 96-well microtiter plates. The enzyme reaction was initiated by adding appropriate substrate (FXa, S-2222). The 96-well plate for fluorescence was prepared with 40 μL of enzyme and 1 μL of each compound. To initiate the enzymatic reaction 50 μL of the factor Xa substrate was added to each well and the plate was read with a fluorescence microplate reader FLx800 (Biotek Inc., Winooski, VT, USA) programmed to read continuously for 30 min at 37 °C, at a wavelength of Ex/Em 490 nm/520 nm. The plate was observed to discard the insoluble compounds and then analyze the inhibition effect of the soluble compounds only. The data obtained was analyzed with Prism v.7 (GraphPad Inc., La Jolla, CA, USA). In the second trial, the ten filtered and selected compounds) were prepared to span four different concentrations: 0.1–10 nM. The IC_50_ was calculated from the mean of triplicates from a dilution series of the compound with Prism v.7 (GraphPad Inc., La Jolla, CA, USA).

### 3.5. In Vitro Coagulation Assays

PT and aPTT assays were performed on an ACLTop (Instrumentation Laboratory) coagulation instrument. Recombiplastin 2G and aPTT-SP liquid were used according to standard procedures. Increasing concentrations of inhibitor ranging from 0.1 to 10 nM or solvent were added to human plasma and incubated for 10 min at 37 °C. Clotting times were measured and compared with those from the appropriate control plasma.

## 4. Conclusions

We have successfully identified a novel FXa inhibitor compound **5** (NSC635393) (4-(3-methyl-4*H*-1,4-benzothiazin-2-yl)-2,4-dioxo-*N*-phenylbutanamide), which shows in vitro activity in the nanomolar range by means of a combined ligand- and structure-based virtual screening approach. In vitro coagulation assays suggest that keto-enol tautomerization of compound **5** (NSC635393) increases protein plasma binding and may reduce FXa inhibition under assay conditions.

## Figures and Tables

**Table 1:** Summary of crystal structures of factor Xa-ligand complexes used in this study.

PDB ID (Reference)	Resolution (Å)	Ligand Structure	RMSD Cluster Center (Å)	RMSD Binding Site (6 Å) ^a^	Cluster ID	Tanimoto Distance ^b^
2VWN [30]	1.61	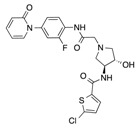	-----	-----	I	0.000
2VWO [30]	1.60	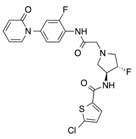	0.435	0.269	I	0.091
2VVC [30]	1.95	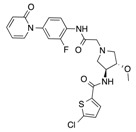	0.320	0.711	I	0.109
2VVU [30]	2.30	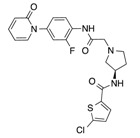	0.327	0.911	I	0.125
2VWL [30]	1.80	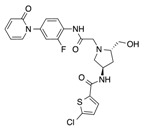	0.137	0.732	I	0.155
2VWM [30]	1.96	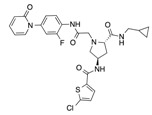	0.338	0.695	I	0.213
2VVV [30]	1.73	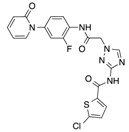	0.301	0.977	I	0.371
2XBX [31]	1.85	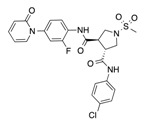	-----	-----	II	0.000
2XC5 [31]	1.70	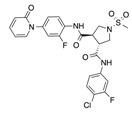	0.121	0.620	II	0.045
2XBW [31]	1.72	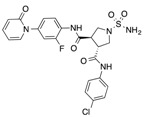	0.194	0.659	II	0.111
2XC0 [31]	2.05	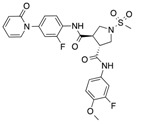	0.120	0.767	II	0.128
2XC4 [31]	1.67	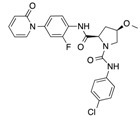	0.358	0.866	II	0.130
2PHB [26]	2.30	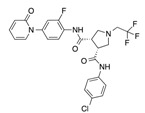	0.416	0.956	II	0.365
2XBV [31]	1.66	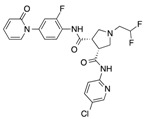	0.193	0.417	II	0.378
2W3K [32]	2.05	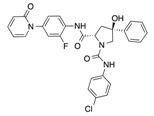	0.377	1.019	II	0.411
4BTI [33]	2.30	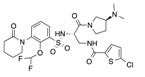	-----	-----	III	0.000
4BTU [33]	2.37	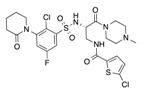	0.440	0.605	III	0.426
4BTT [33]	2.59	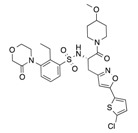	0.530	0.479	III	0.582
2W26 Rivaroxaban [27]	2.08	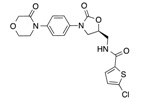	0.276	0.774	III	0.618
2P16 Apixaban [34]	2.30	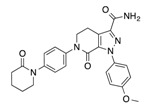	0.290	0.928	III	0.689

^a^ All atoms within 6 Å from the center of mass of ligand alignment, ^b^ 1-Tanimoto Coefficient.

**Table 2:** Summary of biological activity, docking scores, binding energy estimation, and shape/electrostatic similarity scores for selected compounds used in this study. (See Table S1 for full list).

Number	Cluster	NSC Code	FXa Activity Inhibition >50% at 10 μM	FXa Activity Inhibition >50% at 1 μM	ChemGauss4 Score	ΔG Ludi3 (kcal/mol)	Consensus Score	EON ET Combo	EON Rank	ROCS Tanimoto Combo	ROCS Rank
2	3	647716	(+)	(−)	−11.325	−11.06	10	0.733	34	0.541	202
3	2	635553	(+)	(−)	−10.852	−12.26	10	0.919	234	0.541	202
5	2	635393	(+)	(+)	−10.487	−11.25	9	1.095	27	0.632	6
7	1	141296	(+)	(−)	−10.025	−9.42	7	1.274	34	0.686	117
9	1	634395	(+)	(−)	−9.972	−10.12	9	1.164	78	0.593	1187
10	1	351149	(+)	(−)	−9.830	−10.65	7	1.289	29	0.630	500
12	3	371867	(+)	(−)	−9.594	−12.49	6	0.700	72	0.463	2404
9	2	635550	(+)	(−)	−8.880	−12.41	7	0.895	284	0.567	76
22	2	634416	(+)	(−)	−8.737	−9.63	7	1.026	77	0.485	1180
23	2	635142	(+)	(−)	−8.729	−9.87	8	0.989	112	0.481	1329
24	2	646798	(+)	(−)	−7.480	−12.00	7	0.910	250	0.504	617
-	-	Rivaroxaban	(+)	(+)	−13.715	−13.21	10	1.552	1	0.768	7

## Supplementary Materials

The Supplementary Materials are available online.
