# Supplementary material for: Novel FXa Inhibitor Identification through Integration of Ligand- and Structure-Based Approaches
Source: Molecules. 2017 Sep 22;22(10):1588. doi: 10.3390/molecules22101588 (PMC6151700; doi:10.3390/molecules22101588)
Supplement: Supplementary file 1 [file molecules-22-01588-s001.zip › Figuras/Supplementary Figure 1.pdf]

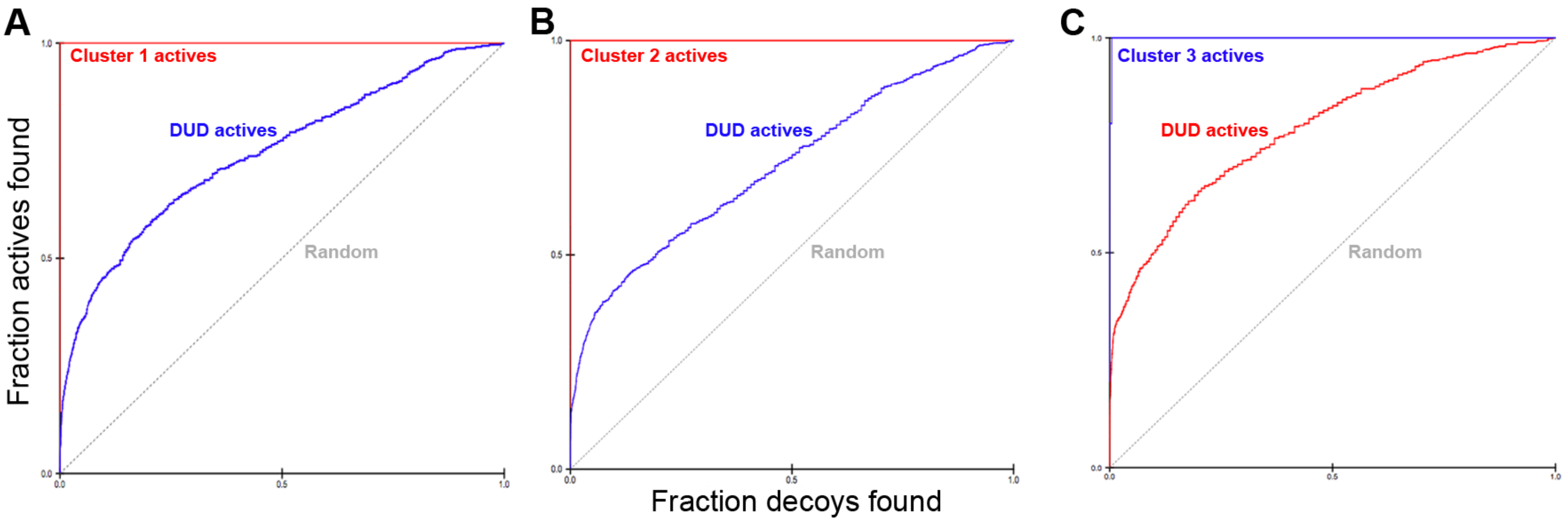

ROCS Statistics*	Cluster 1 Actives (7)	FXa DUD Actives (792)
**AUC**	0.999847	0.743565
95% confidence min	0.999499	0.724189
95% confidence max	1.000000	0.761754
**0.5% Enrichment**	200.000000	30.000189
95% confidence min	200.000000	25.345045
95% confidence max	200.000015	35.400517
**1.0% Enrichment**	100.000000	19.191874
95% confidence min	100.000000	16.416040
95% confidence max	100.000008	21.774193
**2.0% Enrichment**	50.000000	12.311981
95% confidence min	50.000000	10.945273
95% confidence max	50.000004	14.037267

* Based on Tanimoto Combo Score

ROCS Statistics*	Cluster 2 Actives (8)	FXa DUD Actives (792)
**AUC**	0.999847	0.709970
95% confidence min	0.999499	0.688660
95% confidence max	1.000000	0.730982
**0.5% Enrichment**	200.000000	31.396284
95% confidence min	200.000000	26.294821
95% confidence max	200.000015	36.251541
**1.0% Enrichment**	100.000000	18.228065
95% confidence min	100.000000	15.374842
95% confidence max	100.000008	20.838795
**2.0% Enrichment**	50.000000	12.393811
95% confidence min	50.000000	10.704787
95% confidence max	50.000004	13.928113

* Based on Tanimoto Combo Score

ROCS Statistics*	Cluster 3 Actives (5)	Fxa DUD Actives (792)
**AUC**	0.994793	0.790717
95% confidence min	0.999041	0.772904
95% confidence max	1.000000	0.808000
**0.5% Enrichment**	198.666672	51.408276
95% confidence min	200.000000	44.923859
95% confidence max	200.000015	57.985264
**1.0% Enrichment**	99.333336	31.495468
95% confidence min	100.000000	28.328981
95% confidence max	100.000008	34.753662
**2.0% Enrichment**	49.666668	17.344225
95% confidence min	50.000000	15.571775
95% confidence max	50.000004	18.918919

* Based on Tanimoto Combo Score

Supplementary Figure 1. ROCS shape-based query validation statistics for Cluster 1 (**A**), Cluster 2 (**B**) and Cluster 3 (**C**), using the active molecules for each cluster or all available FXa ligand from the DUD-E database
