# Supplementary material for: Novel FXa Inhibitor Identification through Integration of Ligand- and Structure-Based Approaches
Source: Molecules. 2017 Sep 22;22(10):1588. doi: 10.3390/molecules22101588 (PMC6151700; doi:10.3390/molecules22101588)
Supplement: Supplementary file 1 [file molecules-22-01588-s001.zip › Supplementary Table 1.pdf]

Number	Cluster	Structure	NSC Code	FXa Activity Inhibition >50% at 10μM	FXa Activity Inhibition >50% at 1μM	LigScore1	LigScore2	PLP1	PLP2	Jain	PMF	PMF04	ChemGauss4 Score	ΔG Ludi1 (kcal/mol)	ΔG Ludi2 (kcal/mol)	ΔG Ludi3 (kcal/mol)	Consensus Score	EON ET Combo	EON Rank	ROCS Tanimoto Combo	ROCS Rank
1	2	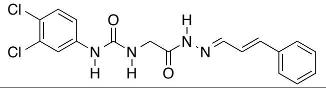	216794	(-)	(-)	-7.03	-13.26	119.19	118.48	4.75	48.53	-16.08	-12.167	-11.69	-8.41	-10.84	7	1.220	2	0.538	233
2	3	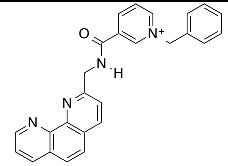	647716	(+)	(-)	1.03	1.47	133.69	123.69	4.39	62.01	14.64	-11.325	-8.45	-7.02	-11.06	10	0.733	34	0.541	202
3	2	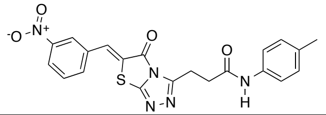	635553	(+)	(-)	2.84	4.44	146.23	127.47	3.85	81.08	46.67	-10.852	-8.97	-7.35	-12.26	10	0.919	234	0.541	202
4	2	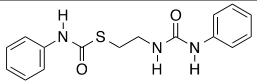	207837	(-)	(-)	2.90	3.84	104.55	101.66	3.14	67.92	32.88	-10.616	-8.24	-6.68	-9.12	8	0.941	192	0.477	1531
**5**	**2**	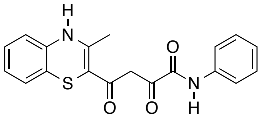	**635393**	**(+)**	**(+)**	**2.84**	**4.23**	**113.21**	**106.49**	**3.83**	**42.72**	**23.17**	**-10.487**	**-6.64**	**-5.90**	**-11.25**	**9**	**1.095**	**27**	**0.632**	**6**
6	2	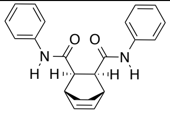	241430	(-)	(-)	4.33	5.03	111.95	103.47	3.23	34.90	-8.99	-10.385	-8.44	-7.20	-8.71	7	1.052	51	0.558	111
7	1	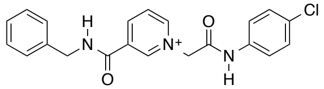	141296	(+)	(-)	4.56	5.40	109.77	106.95	2.65	45.78	8.76	-10.025	-8.10	-6.90	-9.42	7	1.274	34	0.686	117
8	1	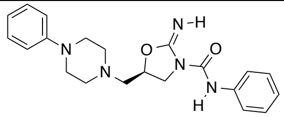	697707	(-)	(-)	3.12	5.17	120.06	106.34	1.99	58.50	43.08	-10.020	-8.13	-6.64	-10.69	9	1.134	97	0.579	1658
9	1	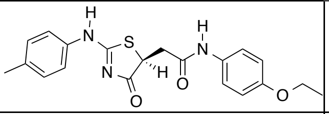	634395	(+)	(-)	4.43	5.68	116.17	105.32	3.91	54.32	23.76	-9.972	-9.38	-7.43	-10.12	9	1.164	78	0.593	1187
10	1	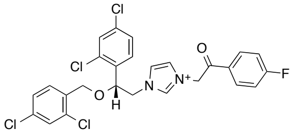	351149	(+)	(-)	-25.76	-40.68	102.58	99.01	3.63	64.65	-22.20	-9.830	-8.65	-7.77	-10.65	7	1.289	29	0.630	500

11	1	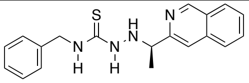	362691	(-)	(-)	2.67	3.61	113.60	110.89	4.51	58.49	31.38	-9.759	-7.35	-6.49	-11.84	8	1.203	58	0.624	579
12	3	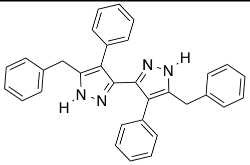	371867	(+)	(-)	-2.21	-5.54	78.89	92.92	5.21	79.96	0.56	-9.594	-10.00	-8.75	-12.49	6	0.700	72	0.463	2404
13	1	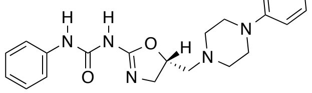	697709	(-)	(-)	0.15	-1.26	88.92	80.25	5.07	57.36	38.36	-9.475	-10.06	-7.87	-11.03	6	1.427	2	0.645	332
14	2	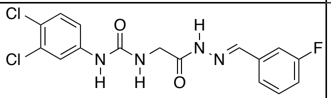	216793	(-)	(-)	-4.97	-11.18	92.04	95.77	4.08	69.58	9.73	-9.467	-10.11	-7.70	-10.23	8	1.218	3	0.476	1609
15	2	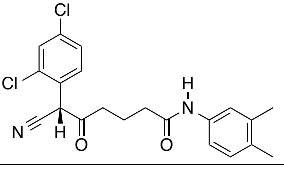	618787	(-)	(-)	2.30	2.09	100.67	102.17	3.19	60.28	8.92	-9.299	-7.12	-6.01	-8.30	7	0.967	145	0.579	55
16	2	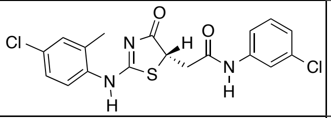	633945	(-)	(-)	4.73	5.24	104.01	97.30	2.68	62.46	18.67	-9.150	-8.51	-6.60	-9.12	8	1.046	54	0.463	2418
17	2	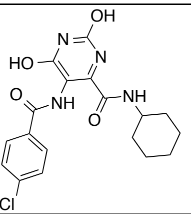	624948	(-)	(-)	3.91	3.55	116.05	109.66	6.17	45.45	0.76	-9.135	-10.49	-8.26	-10.58	10	1.133	12	0.479	1425
18	1	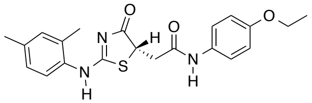	634412	(-)	(-)	2.72	2.88	111.52	109.32	3.55	58.97	28.07	-9.110	-9.75	-7.90	-10.45	9	1.176	71	0.598	1091
19	2	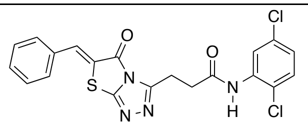	635550	(+)	(-)	-2.41	-4.70	101.24	96.70	3.48	83.02	-8.18	-8.880	-9.14	-7.58	-12.41	7	0.895	284	0.567	76

20	2	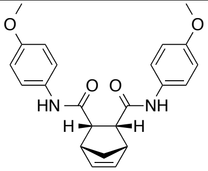	241421	(-)	(-)	3.26	3.41	125.49	120.52	5.59	51.88	4.02	-8.829	-9.30	-7.32	-9.80	10	1.098	24	0.630	7
21	1	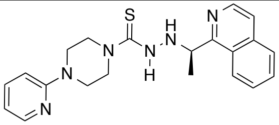	353896	(-)	(-)	-3.36	-5.93	108.69	107.10	3.93	74.56	24.73	-8.790	-8.78	-7.06	-11.20	8	1.341	12	0.576	1800
22	2	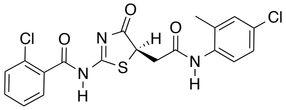	634416	(+)	(-)	-7.74	-12.77	110.25	110.45	2.12	56.81	12.90	-8.737	-8.67	-7.05	-9.63	7	1.026	77	0.485	1180
23	2	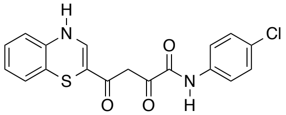	635142	(+)	(-)	4.71	5.57	112.52	114.50	3.16	46.51	10.25	-8.729	-8.17	-6.52	-9.87	8	0.989	112	0.481	1329
24	2	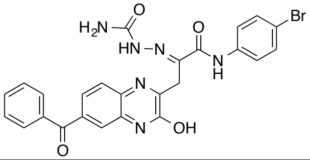	646798	(+)	(-)	-8.92	-16.56	102.96	106.48	2.61	51.77	8.02	-7.480	-10.46	-8.33	-12.00	7	0.910	250	0.504	617
25	2	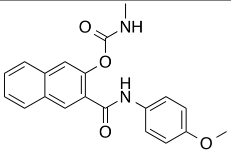	204570	(-)	(-)	2.56	1.16	103.15	95.42	2.94	7.87	-20.75	-7.453	-9.64	-7.40	-6.83	6	0.988	114	0.541	204
26	2	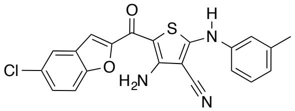	309892	(-)	(-)	2.10	2.70	97.72	98.56	3.41	44.77	-1.03	-7.289	-9.07	-7.16	-11.73	8	0.959	163	0.457	2976
27	2	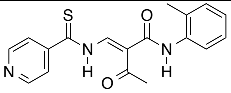	658322	(-)	(-)	1.53	1.34	105.10	102.90	2.74	40.76	-0.84	-7.165	-9.48	-7.57	-10.06	7	0.908	255	0.468	2049
28	1	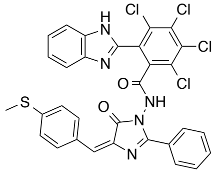	716348	(-)	(-)	-12.18	-21.74	112.86	112.60	3.36	78.42	15.68	-5.123	-11.96	-9.08	-13.54	7	1.173	74	0.581	1568

29	2	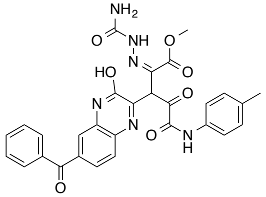	649836	(-)	(-)	-2.74	-8.38	98.40	111.21	3.48	42.17	-2.04	-4.725	-10.72	-8.51	-11.32	6	0.926	224	0.474	1728
30	3	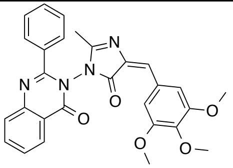	657360	(-)	(-)	-0.74	-2.43	70.45	71.01	3.92	78.57	44.24	-3.579	-8.52	-6.72	-11.14	7	0.704	67	0.463	2380
